# Autophagy Mediates Interleukin-1β Secretion in Human Neutrophils

**DOI:** 10.3389/fimmu.2018.00269

**Published:** 2018-02-19

**Authors:** Leonardo Iula, Irene A. Keitelman, Florencia Sabbione, Federico Fuentes, Mauricio Guzman, Jeremías Gastón Galletti, Pehuén Pereyra Gerber, Matías Ostrowski, Jorge R. Geffner, Carolina C. Jancic, Analía S. Trevani

**Affiliations:** ^1^Laboratorio de Inmunidad Innata, Instituto de Medicina Experimental (IMEX)––CONICET, Academia Nacional de Medicina, Buenos Aires, Argentina; ^2^Instituto de Investigaciones Biomédicas en Retrovirus y SIDA (INBIRS), CONICET, Facultad de Medicina, Universidad de Buenos Aires, Buenos Aires, Argentina; ^3^Departamento de Microbiología, Parasitología e Inmunología, Facultad de Medicina, Universidad de Buenos Aires, Buenos Aires, Argentina

**Keywords:** neutrophil, autophagy, interleukin-1β, unconventional secretion, serine proteases

## Abstract

Interleukin-1β (IL-1β), a major pro-inflammatory cytokine, is a leaderless cytosolic protein whose secretion does not follow the classical endoplasmic reticulum-to-Golgi pathway, and for which a canonical mechanism of secretion remains to be established. Neutrophils are essential players against bacterial and fungi infections. These cells are rapidly and massively recruited from the circulation into infected tissues and, beyond of displaying an impressive arsenal of toxic weapons effective to kill pathogens, are also an important source of IL-1β in infectious conditions. Here, we analyzed if an unconventional secretory autophagy mechanism is involved in the exportation of IL-1β by these cells. Our findings indicated that inhibition of autophagy with 3-methyladenine and Wortmannin markedly reduced IL-1β secretion induced by LPS + ATP, as did the disruption of the autophagic flux with Bafilomycin A1 and E64d. These compounds did not noticeable affect neutrophil viability ruling out that the effects on IL-1β secretion were due to cell death. Furthermore, VPS34IN-1, a specific autophagy inhibitor, was still able to reduce IL-1β secretion when added after it was synthesized. Moreover, siRNA-mediated knockdown of ATG5 markedly reduced IL-1β secretion in neutrophil-differentiated PLB985 cells. Upon LPS + ATP stimulation, IL-1β was incorporated to an autophagic compartment, as was revealed by its colocalization with LC3B by confocal microscopy. Overlapping of IL-1β-LC3B in a vesicular compartment peaked before IL-1β increased in culture supernatants. On the other hand, stimulation of autophagy by cell starvation augmented the colocalization of IL-1β and LC3B and then promoted neutrophil IL-1β secretion. In addition, specific ELISAs indicated that although both IL-1β and pro-IL-1β are released to culture supernatants upon neutrophil stimulation, autophagy only promotes IL-1β secretion. Furthermore, the serine proteases inhibitor AEBSF reduced IL-1β secretion. Moreover, IL-1β could be also found colocalizing with elastase, suggesting both some vesicles containing IL-1β intersect azurophil granules content and that serine proteases also regulate IL-1β secretion. Altogether, our findings indicate that an unconventional autophagy-mediated secretory pathway mediates IL-1β secretion in human neutrophils.

## Introduction

Neutrophils are the most numerous leukocytes in human circulation. These cells represent the first line of cellular defense against bacterial and fungal infections. They also often comprise more than 70% of the leukocyte-infiltrating viral infection sites, playing roles which are currently being defined (1). Neutrophils are crucial in the response to a broad range of clinically relevant pathogens, a fact underscored by the occurrence of severe, and often fatal, infections in patients with congenital neutrophil deficiencies (2). These cells engulf and kill bacteria by producing reactive oxygen species into a phagocytic vacuole (3). They also contain different granule subsets that are mobilized by stimulation, and fuse with the cell membrane or the phagosomal membrane, resulting in exocytosis and/or exposure of soluble and membrane-bound proteins essential not only for phagocytosis, and elimination of microorganisms but also during neutrophil–endothelial interaction and extravasation (4).

Neutrophils have been traditionally considered within the innate immune response setting for its antimicrobial capacity. However, recent studies performed with highly purified neutrophils have shown that these cells also produce and release different cytokines that may potentially influence the course of an immune response (5). Among these cytokines is interleukin-1β (IL-1β), a key pro-inflammatory cytokine that exerts pleiotropic effects on both the innate and adaptive immune system (6, 7). *In vitro*, neutrophils usually make lower levels of this cytokine than monocytes/macrophages do on a per-cell basis; however, *in vivo*, neutrophils constitute the majority of infiltrating cells in inflamed tissues and often outnumber mononuclear leukocytes by one to two orders of magnitude (2). Thus, under those circumstances, the contribution of neutrophil-derived IL-1β to acute and chronic inflammatory conditions might be of foremost importance (8, 9). In fact, previous studies showed that neutrophils but not monocytes/macrophages were the predominant source of IL-1β at a site of *Staphylococcus aureus* cutaneous infection and demonstrated that neutrophil-derived IL-1β was critical for abscess formation and host defense (8). Other studies using models of group-B streptococcus-induced peritoneal inflammation found that locally recruited neutrophils significantly contribute to IL-1β production (10). Further studies also indicated that neutrophils are the major source of IL-1β in a *Streptococcus pneumoniae* corneal infection model (11) and a relevant source of IL-1β in response to acute *Pseudomonas aeruginosa* infection during acute pneumonia and peritonitis (12).

Interleukin-1β is a multifunctional and one of the most potent pro-inflammatory cytokines (13). It is synthesized in the cytoplasm as a precursor, pro-IL-1β, which has to be proteolytically processed to acquire biological activity. We have previously demonstrated that human neutrophil IL-1β processing is dependent of caspase-1 and the neutral proteases elastase and/or proteinase-3. We also reported that NADPH oxidase-derived ROS are dispensable for neutrophil inflammasome activation but are required for IL-1β secretion (7). Unlike proteins endowed with the leader (N-terminal signal) peptides, IL-1β is a leaderless cytosolic protein which cannot enter the conventional secretory pathway normally operating *via* the endoplasmic reticulum and the Golgi apparatus. Several pathways have been proposed to explain IL-1β secretion in other myeloid cells. However, the definition of these pathways still remains controversial (13). Autophagy (macroautophagy) has been often defined as a degradative process and a tributary of the lysosomal pathway, which contributes to remove defunct or disused organelles, particulate targets and invading microbes (14). However, recent studies suggested that autophagy could be involved in the secretion of leaderless proteins like IL-1β (15), even though other studies ascribed to autophagy a role in dampening IL-1β activation by the inflammasome (16). Probably, contributing to these contrasting findings is the fact that IL-1β is subjected to regulation at the level of transcription, translation, processing, and secretion; all mechanisms which could diverge among different cell types (6, 7). Considering that stimuli like LPS that promote IL-1β secretion also induce autophagy and the fact that no previous studies have analyzed the pathways involved in IL-1β exportation from human neutrophils, here we aim to determine whether an unconventional secretory autophagy mechanism is involved in the secretion of IL-1β by these cells.

## Materials and Methods

The experimental protocols performed have been approved by the Biosafety and Research Review boards of the “Instituto de Medicina Experimental-CONICET-Academia Nacional de Medicina” and the Ethical Committee of the “Institutos de la Academia Nacional de Medicina.” The methods were carried out in accordance with the approved guidelines.

### Reagents and Materials

RPMI 1640 culture medium, Earle’s Balanced Salt Solution (EBSS), and TMB substrate were purchased from Thermo Fisher Scientific Life Technologies (MA, USA). Fetal bovine serum (FBS) and bovine serum albumin were purchased from Internegocios (Buenos Aires, Argentina). Ficoll-Paque was purchased from GE Healthcare (Munich, Germany). BD OptEIA™ Human IL-1β ELISA Set II and Human IL-8/CXCL8 ELISA Set were purchased from BD Biosciences (Franklin Lakes, NJ, USA). Quantikine Human Pro-IL-1β/IL-1F2 Immunoassay was purchased from R&D (Minneapolis, MN, USA). Secondary antibodies were purchased from Jackson Immunoresearch Laboratories (West Grove, PA, USA): Alexa Fluor^®^ 647 AffiniPure F(ab’)_2_ Fragment Goat Anti-Rabbit IgG (H + L), cat. #111-606-144; Alexa Fluor^®^ 488 AffiniPure F(ab’)_2_ Fragment Goat Anti-Rabbit IgG (H + L) cat. #111-546-144; DyLight 549 conjugated AffiniPure F(ab’)_2_ Fragment Goat Anti-mouse IgG (H + L), cat. #115-506-062. TO-PRO-3 was obtained from Life Technologies (Carlsbad, CA, USA). Phycoerythrin-conjugated anti-CD14 antibody was purchased from eBioscience (San Diego, CA, USA). Aqua-Poly/Mount Coverslipping Medium was purchased from Polysciences (Warrington, PA, USA). Rabbit polyclonal antibody anti-LC3B cat. #sc28266 was from Santa Cruz Biotechnology (Dallas, TX, USA); mouse monoclonal AS10 anti-IL-1 beta antibody cat. #LS-C26495 and cat. #552289 were purchased from LifeSpan BioScience and BD Pharmingen, respectively; and purified mouse IgG1, κ isotype control cat. #555746 and rabbit polyclonal IgG was purchased from Jackson Immunoresearch. VPS34-IN1, E64d, and 3-methyladenine (3-MA) were purchased from Cayman. All other chemicals employed were purchased from Sigma Aldrich (St. Louis, MO, USA).

### Human Neutrophil Isolation

Neutrophils were isolated from heparinized human blood from healthy donors who gave written informed consent, by centrifugation on Ficoll-Paque, dextran sedimentation, and hypotonic lysis (7). Cells were suspended at 6 × 10^6 ^/mL in RPMI 1640 supplemented with penicillin (100 U/mL), streptomycin (100 µg/mL), and 10% FBS. After isolation, neutrophil preparations were stained with an anti-CD14-PE antibody and analyzed with a FACSCalibur cytometer (Beckton Dickinson, San Jose, CA, USA) to guarantee that monocyte contamination was <0.5% (Figure S1 in Supplementary Material). Cells were used immediately after isolation.

### Neutrophil Stimulation

Neutrophils were treated for 2 h with or without 250 ng/mL LPS from *Escherichia coli* O111:B4. Then, they were stimulated or not with 2.5 mM ATP and cultured for the times specified. Where indicated, before LPS stimulation, cells were pretreated for 30 min with 3 MA (5 mM) or Wortmannin (100 nM). Alternatively, at 1 h (AEBSF) or 3 h post-LPS stimulation cells were treated with AEBSF (1 mM), Bafilomycin A1 (Baf A1; 100 nM), or E64D (10 µM). After culture, cell supernatants, and where indicated also the cell pellets, were recovered and IL-1β, pro-IL-1β, and IL-8 concentrations were quantitated by ELISA. In cell pellets, viability was determined by annexin V-FITC/propidium iodide (PI) staining and flow cytometry analysis. Alternatively, at different time points post-LPS stimulation, cell pellets were fixed with 4% paraformaldehyde (PFA), and processed for confocal laser scanning microscopy (CLSM) or flow cytometry. In some experiments, where indicated, the time points of addition of inhibitors and evaluation of the results were modified according to the aims of the assay. In a set of experiments, at 3.5 h post-LPS stimulation, supernatants were removed and replaced by complete medium (mock starved) or EBSS (starvation).

### ATG5 Knockdown

PLB985 cells (kindly provided by Dr. Mary Dinauer; Washington University Medical School, St. Louis, MO, USA) were differentiated to granulocytes by exposure to 0.5% dimethylformamide for 5 days, as previously described (7). Knockdown of ATG5 was performed by employing a pool of four siGENOME Human ATG5 (9474) siRNA-SMARTpool [M-004374-04-0005 from Dharmacon (Lafayette, CO, USA)] or a non-targeting siRNA (scramble) used as a control. PLB985 cells were transfected in the third day of differentiation using the Amaxa system (Lonza), according to the manufacturer’s instructions. ATG5 expression was determined using an anti-ATG5 polyclonal antibody (cat. #10181-2-AP, Proteintech Group Inc., Rosemont, IL, USA) by flow cytometry.

### Intracellular Immunostainings and Confocal Laser Scanning Microscopy Acquisition

After fixation with PFA 4% for 30 min, cells were blocked with PBS-glycine (0.1 M) for 15 min, permeabilized with chilled acetone (−20°C) for 7 min, rehydrated with PBS and blocked with PBS supplemented with 5% goat serum overnight at 4°C. Then, neutrophils were incubated with the primary antibodies in blocking buffer for 1 h at room temperature, washed, and then incubated with the corresponding secondary antibodies for 1 h at room temperature. In some experiments TO-PRO-3 was added for nuclei staining. Then cells were washed, cytospinned, mounted with AcquaPolymount mounting medium, and stored at 4°C until microscopy examination. Image acquisition was performed by using a FluoView FV1000 confocal microscope (Olympus, Tokyo, Japan) equipped with a Plapon 60X/1.42 objective. Images were analyzed with ImageJ software (NIH) and fluorescence was quantitated. Some CLSM experiments were performed by seeding neutrophils on poly-l-lysine coated Lab-Tek chambers (Nalge Nunc International, New York, NY, USA).

### Automated Image Analysis

Images were analyzed using Fiji software and macros for automatized image quantification were designed. In all cases, the values were obtained individually per cell using regions of interest (ROIs) established on the IL-1β corresponding image. A low threshold that included the fluorescence of the whole cell area was applied for the generation of a mask image. Fields of low cellular density were selected for imaging; the tool watershed was applied for separation of attached cells in the mask image and to generate individual ROIs corresponding to each cell. Intensity of IL-1β and LC3B signal in the vesicular vs. the cellular compartment was quantified. For each image, a background value was subtracted. This value was obtained by averaging the intensity inside the nuclear region. Two masks were created: one for the vesicular and another for the cellular compartment. The vesicular threshold was arbitrarily established for IL-1β images. Both masks were applied to the background subtracted-images, and the integrated density of the intensity in both compartments was measured in each cell.

For the colocalization of LC3B and IL-1β, a threshold was established in each image and the determination of the Manders’ M1 and M2 coefficients of individual cells were performed using the coloc2 plugin. The colocalizing areas were measured and expressed as percentage of the total area of the cell.

### Flow Cytometry Analysis

In some experiments, after immunostaining, cell fluorescence was determined by flow cytometry using a FACscalibur BD Pharmingen flow cytometer or a Partec Cyflow cytometer. Data were analyzed by using the software FlowJo.

### Statistical Analysis

Statistical analysis in each experiment was performed by two-way ANOVA followed by Bonferroni’s multiple comparisons test. Otherwise the Mann–Whitney *U* test was used for the analysis of two unpaired groups of confocal images, or alternatively, a generalized linear model (GLZ) with ANOVA for the kinetic analysis. The statistical calculations were performed using GraphPad Prism version 6.00 for Windows, GraphPad Software, La Jolla, CA, USA or InfoStat software for GLZ analysis. Statistical significance was defined as *p* < 0.05.

## Results

**Figure 1:**
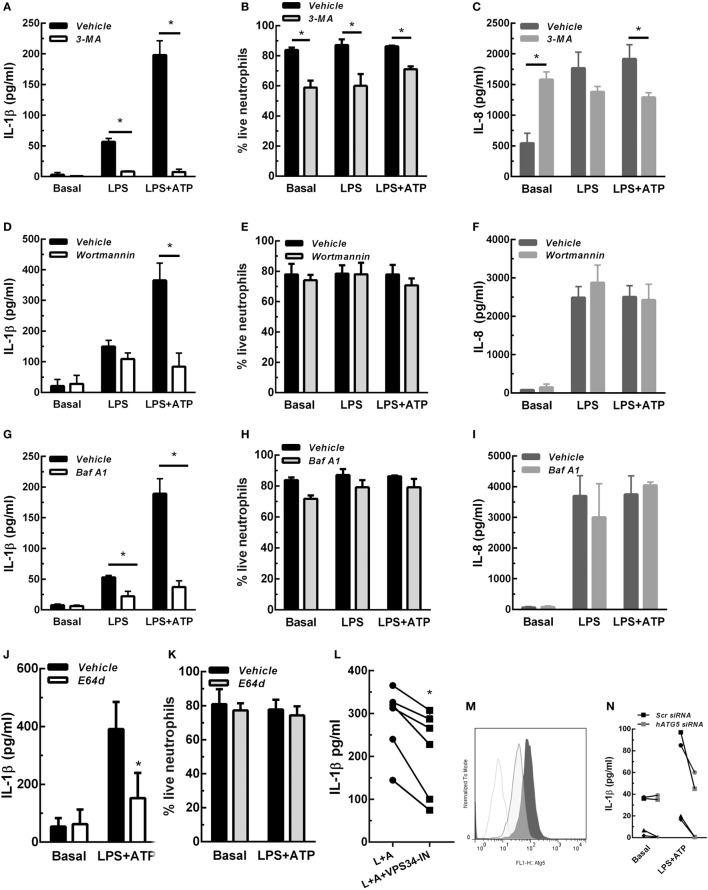
Concentrations of interleukin-1β (IL-1β) **(A,D,G,J,L)** and IL-8 **(C,F,I)** in culture supernatants of neutrophils stimulated with LPS or LPS + ATP in the absence (vehicle) or presence of 3-methyladenine (3-MA) [5 mM; **(A)** and **(C)**] or Wortmannin [50 nM; **(D,F)**] added 30 min before LPS stimulation; Baf A1 [100 nM; **(G,I)**] or E64d [10 µM; **(J)**] added at 3. 5 h; or VPS34-IN1 [1 µM; **(L)**] added at 3 h post-LPS stimulation. **(B,E,H,K)** Viability of neutrophils subjected to treatment with the indicated inhibitors evaluated by annexin V/propidium iodide staining and flow cytometry. Data represent the mean ± SEM of 3–6 experiments performed in triplicate (for cytokines) and duplicate (for viability). **(M)** Histograms represent ATG5 expression in scramble- (Scr; dark gray tinted) or ATG5-siRNA transfected (light gray tinted) neutrophil-differentiated PLB985 cells and isotype control (gray line). **(N)** Graph depicts IL-1β concentrations in culture supernatants of these cells stimulated or not with LPS + ATP. Black symbols, Scr siRNA; gray symbols, hATG5 siRNA. Two-way ANOVA followed by Bonferroni’s multiple comparisons test. *p < 0.05.

In previous studies we determined that upon stimulation with LPS and LPS + ATP, human neutrophils produce and release IL-1β (7). These studies also indicated that similar IL-1β levels were detected extracellularly at either 5- or 18 h post-stimulation with LPS + ATP. Thus, to establish the shortest stimulation period that allowed us to determine whether autophagy is required for IL-1β secretion by human neutrophils, we first evaluated IL-1β secretion at 3-, 4-, and 5 h post-stimulation. Results indicated that IL-1β secretion was maximal at 5 h post-stimulation with LPS + ATP (Figure S2 in Supplementary Material). However, we also detected donor specific variability as some samples exhibited earlier responses, probably due to differences in donor responsiveness to ATP, as was previously reported (17). We then reasoned that if autophagy is involved in IL-1β secretion, this mechanism should be evidenced before secretion has taken place. Thus, we then evaluated if LPS + ATP triggers neutrophil autophagy. To this aim, we took advantage of the fact that in cells undergoing autophagy, the cytoplasmic protein LC3B (LC3B-I) conjugates with phosphatidylethanolamine in the membrane of autophagosomes (LC3B-II) and can be detected by CLSM. In agreement with an increase in autophagy, at 3 h post-stimulation with LPS + ATP, neutrophils exhibited enhanced punctuated LC3B staining compared with unstimulated cells (basal; Figure S3A in Supplementary Material). We then evaluated the effect of Baf A1, a compound which disrupts autophagic flux and as a consequence impairs LC3B-II autolysosomal degradation (18). We observed an augment of LC3B + punctae at 3 h post-stimulation in the presence of Baf A1 (Figure S3A in Supplementary Material). These findings confirmed that LPS + ATP induced an increase in autophagy and was not blocking the autophagic flux (19). Quantification of the images indicated that the percentage of vesicular LC3B increased in cells stimulated with LPS + ATP for 3 h compared with the percentage in cells that remained unstimulated during the whole culture period (Figures S3B,C in Supplementary Material). As neutrophils are terminally differentiated cells that cannot be transfected, we took advantage of distinct compounds that inhibit different autophagy steps to address the role of this mechanism in IL-1β secretion. We first evaluated the impact of 3-MA and Wortmannin, two autophagy inhibitors that block an early stage of the process by inhibiting the PtdIns 3-kinase. We found that 3-MA nearly abrogated IL-1β release induced by 5 h stimulation with LPS and LPS + ATP, while Wortmannin markedly reduced that induced by LPS + ATP (Figures 1A,D). Importantly, the decrease in IL-1β secretion mediated by these inhibitors cannot be ascribed to a cytotoxic effect. In fact, 3-MA almost abrogated IL-1β secretion but slightly reduced cell viability (Figure 1B; Figure S4 in Supplementary Material), an effect that has been previously reported (20), while Wortmannin did not modify it (Figure 1E; Figure S4 in Supplementary Material). To rule out that these compounds were exerting an overall inhibitory effect on protein secretion, we also determined their impact on the secretion of IL-8, a cytokine that is released following the canonical ER-Golgi pathway. While Wortmannin did not affect IL-8 secretion (Figure 1F), 3-MA slightly decreased it (Figure 1C), even though this effect was negligible as compared with that exerted on IL-1β secretion. Of mention, 3-MA did not affect IL-1β synthesis, and as expected, induced intracellular IL-1β accumulation, even though this effect was transient (Figure S5 in Supplementary Material). In additional assays, we evaluated the impact of blocking the autophagic flux with Baf A1 on IL-1β secretion. This compound also inhibited IL-1β secretion (Figure 1G), and its effect was not due to a reduction in neutrophil viability (Figure 1H; Figure S4 in Supplementary Material) and was not exerted on IL-8 secretion (Figure 1I). We made similar findings with E64d, a cysteine proteases inhibitor that prevents autolysosomal degradation (Figures 1J,K; Figure S4 in Supplementary Material). We also found that VPS34-IN1, a specific inhibitor of class III PI3K that blocks an early stage of autophagy, was still able to partially reduce IL-1β secretion if added at 3 h post-LPS stimulation (Figure 1L). Taken together, these results suggest that a non-obstructed autophagic flux is required for IL-1β secretion. This conclusion was strengthened by results of additional assays performed with neutrophil-differentiated PLB985 cells, in which the expression of the essential component of the autophagic pathway ATG5 was reduced by siRNA knockdown (Figure 1M). As expected, ATG5 knockdown markedly reduced the secretion of IL-1β induced by LPS + ATP (Figure 1N), but not IL-1β synthesis (Figure S6 in Supplementary Material).

**Figure 2:**
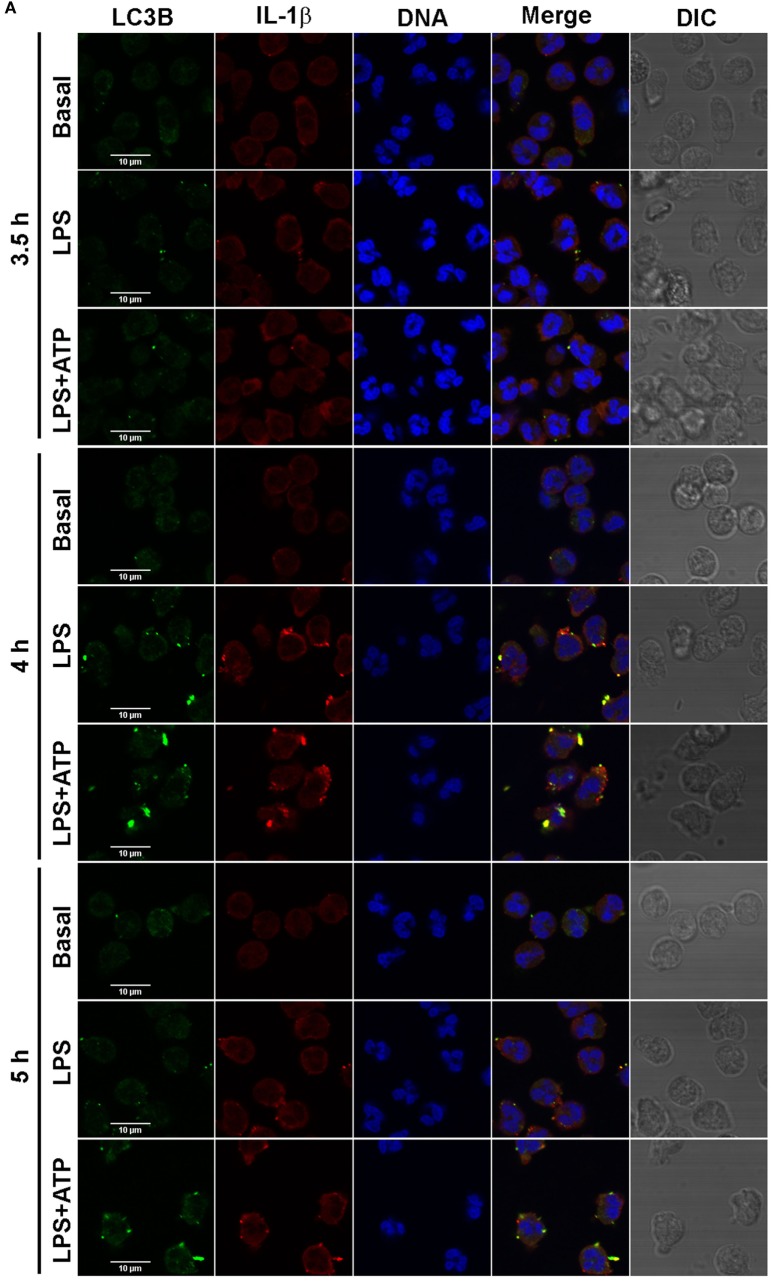

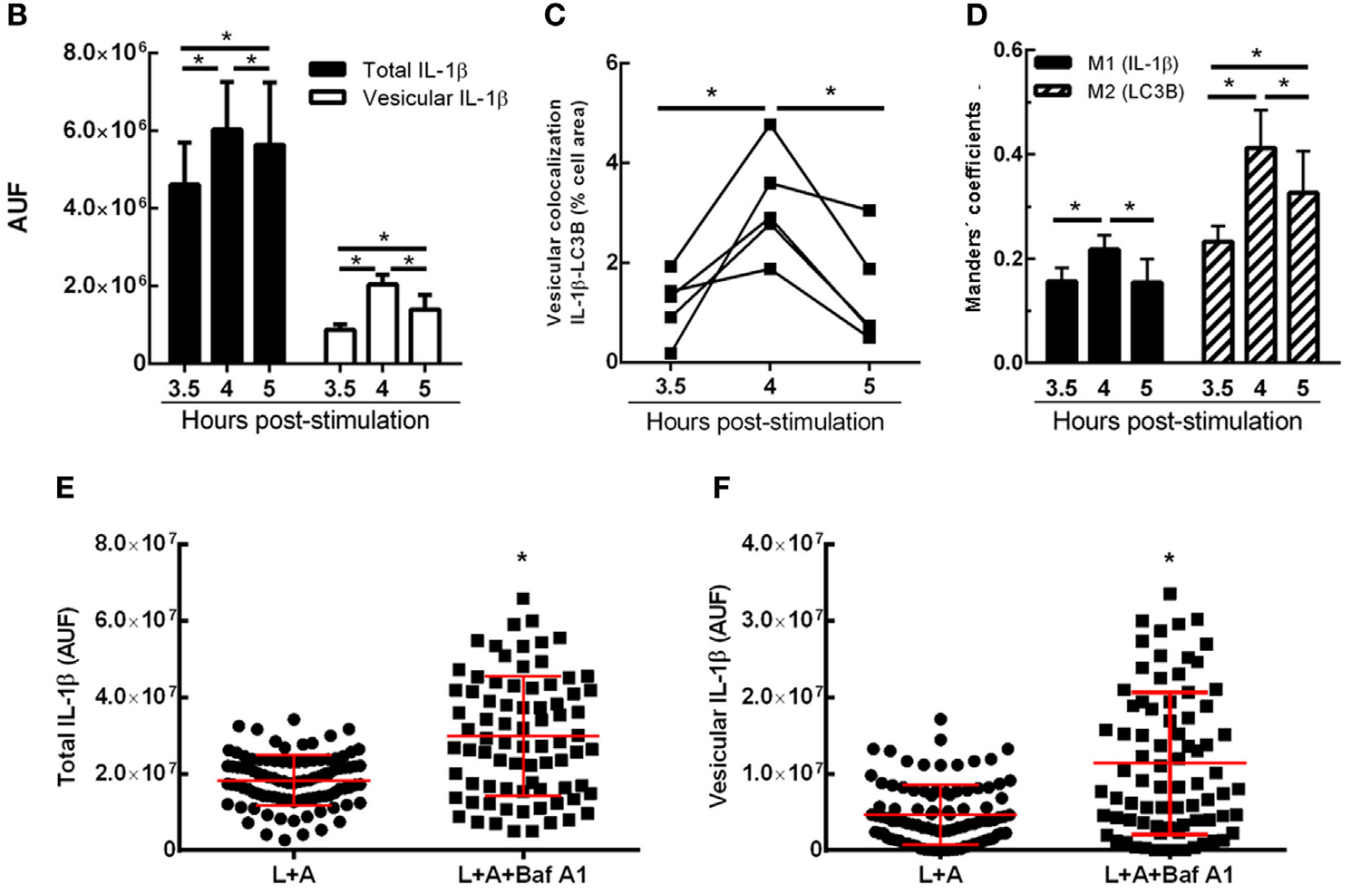
Kinetic of interleukin-1β (IL-1β) localization upon stimulation of neutrophils with LPS or LPS + ATP. Neutrophils were stimulated with LPS and 2 h later were treated or not with ATP. At 3.5-, 4-, and 5 h post-LPS stimulation, cells were fixed, permeabilized, and stained with specific antibodies anti-IL-1β (red) and LC3B (green). Images were acquired with a confocal microscope **(A)** and quantifications **(B–D)** were performed by using specific macro with Fiji software. **(A)** Representative images of 3–5 experiments. **(B)** Fluorescence corresponding to total and vesicular IL-1β/cell expressed in arbitrary units of fluorescence (AUF). **(C)** Percentage of the total cell area with vesicular overlapping of the signals corresponding to IL-1β and LC3B. **(D)** Manders’ colocalization coefficients for IL-1β (M1) and LC3B (M2). **(B–D)** Values were derived from five independent experiments with at least 35 cells analyzed at each time point for each experiment. **(E,F)** Scatter plot depicts the fluorescence of individual cells imaged by confocal microscopy corresponding to total **(E)** and vesicular **(F)** IL-1β/cell expressed in AUF of a representative experiment of two. Red bars indicate the mean ± SEM values of 98 (LPS + ATP) and 78 (LPS + ATP + Baf A1) cells analyzed. A generalized linear model (GLZ) with ANOVA was applied for the kinetic analysis **(B–D)**. * *p* < 0.05.

To get insight if autophagy is required for translocation of cytosolic IL-1β to the inside of a vesicular compartment that could be then conducted by membranes fusion to the extracellular medium, we then determined whether IL-1β is incorporated to autophagic vesicles. To this end, we analyzed the intracellular distribution of IL-1β and evaluated if this cytokine colocalizes with the autophagy marker LC3B by immunostaining and CLSM after 3.5-, 4-, and 5 h post-stimulation with LPS + ATP. As shown in the images of a representative experiment in Figure 2A, and from quantification of images acquired of five independent experiments (Figure 2B), at 3.5 h post-stimulation, IL-1β distribution was both cytoplasmic and vesicular. Some IL-1β+ vesicles overlapped with the LC3B signal (Figures 2A,C). At 4 h post-stimulation, higher levels of IL-1β were observed, with greater amounts of this cytokine showing a vesicular distribution (Figures 2A,B). Moreover, much of these IL-1β-containing vesicles were also positive for LC3B staining (Figures 2A,C). At 5 h post-stimulation, a reduction in vesicular IL-1β as well as in vesicular IL-1β/LC3B colocalization was observed (Figures 2A,B), in agreement with the fact that by this time point greater amounts of IL-1β are secreted and can be found in culture supernatants (Figure S2 in Supplementary Material). However, at this time point, some IL-1β was still found colocalizing with LC3B (Figure 2A). To quantify the degree of colocalization between IL-1β and LC3B, we performed an image analysis by using the Manders’ coefficients. The values of these coefficients range from 0 to 1, for no colocalization to perfect colocalization, respectively. Image analysis showed that Manders’ coefficients significantly increased at 4 h post-stimulation compared with those determined at 3.5 h, indicating that a greater amount of both total LC3B and IL-1β were found colocalizing by this time (Figure 2D). At 5 h post-stimulation, Manders’ coefficients underwent a reduction as compared with that determined at 4 h, which was in agreement with the fact that part of IL-1β was secreted by this time point (Figure 2D; Figure S2 in Supplementary Material). Of mention, as control, we also evaluated if IL-8 colocalized with LC3B. As expected for a conventionally secreted cytokine, IL-8 did not colocalize with LC3B. In fact, IL-8 was hardly found under our experimental conditions, and monensin had to be added to neutrophil cultures in order to detect it (Figure S7 in Supplementary Material).

We then performed additional assays with Baf A1, a compound which not only inhibits V-ATPase-dependent autolysosome acidification but also disrupts the autophagic flux (18). Thus, we reasoned that if Baf A1 is added at early times points post-LPS stimulation (2.15 h), IL-1β should be accumulated. As presumed, total intracellular and vesicular IL-1β levels were increased upon Baf A1 treatment when evaluated at 3 h post-LPS stimulation (Figures 2E,F). These findings are in agreement with the notion that both incorporation of cytosolic IL-1β to vesicles is hampered if the autophagic flux is obstructed, and potential autolysosome degradation by acidic proteases is blocked.

**Figure 3:**
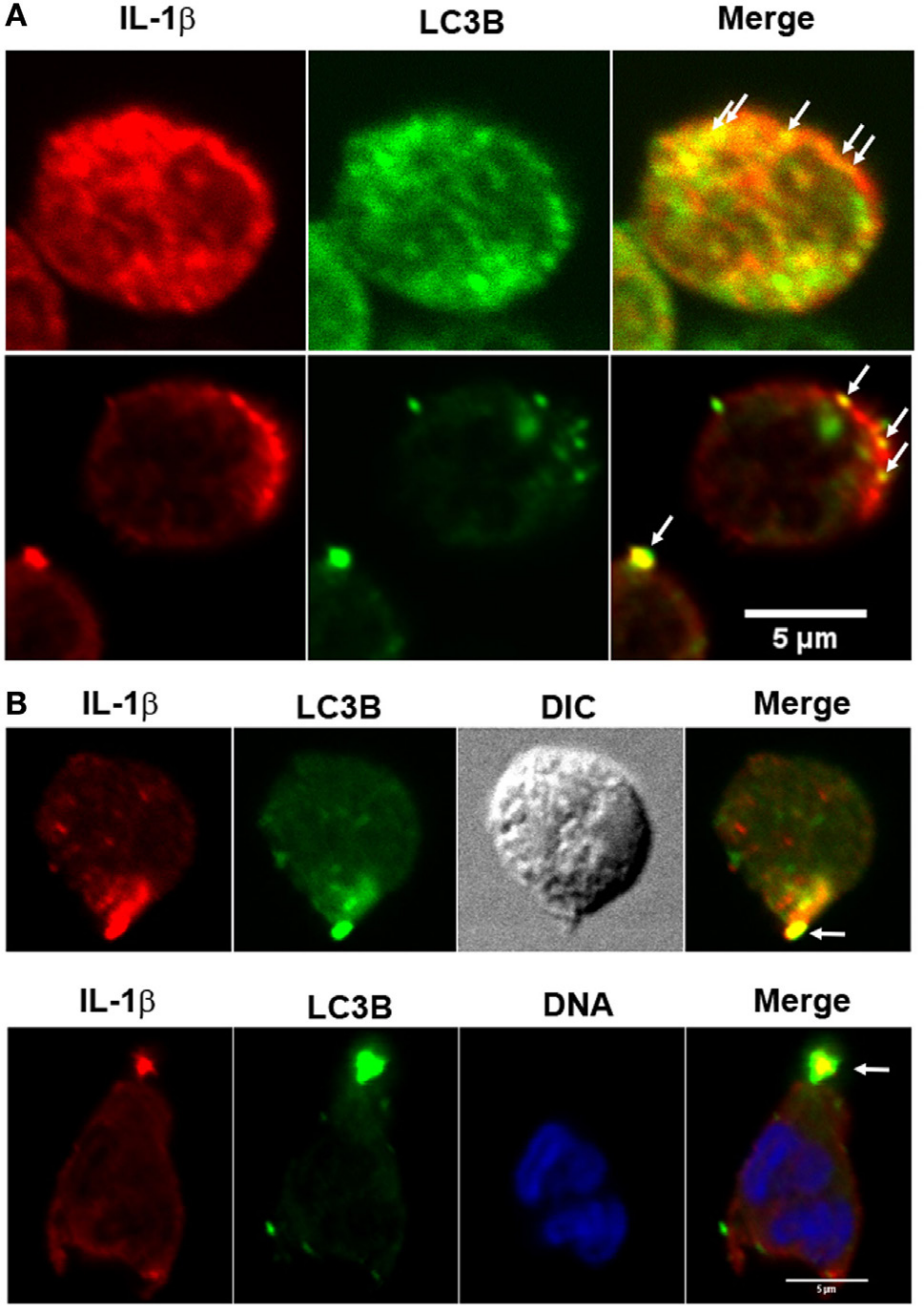
Fluorescence distribution of interleukin-1β (IL-1β) and LC3B in LPS + ATP-stimulated neutrophils. Neutrophils were fixed at 4 h post-LPS stimulation, permeabilized, and stained with specific antibodies anti-IL-1β (red) and LC3B (green). Blue signal represents DNA stained with TO-PRO-3. Images were acquired with a confocal microscope. Arrows indicate compartments with overlapping fluorescent signals near the cell membrane **(A)** and in a cell pole **(B)**. Images are representative of experiments with 10 different donors.

In accordance with the hypothesis that IL-1β is secreted from an autophagic compartment, we were able to detect autophagy vesicles containing IL-1β in discrete places near the cell membrane (Figure 3A) and in occasions in a pole of the cell (Figure 3B).

**Figure 4:**
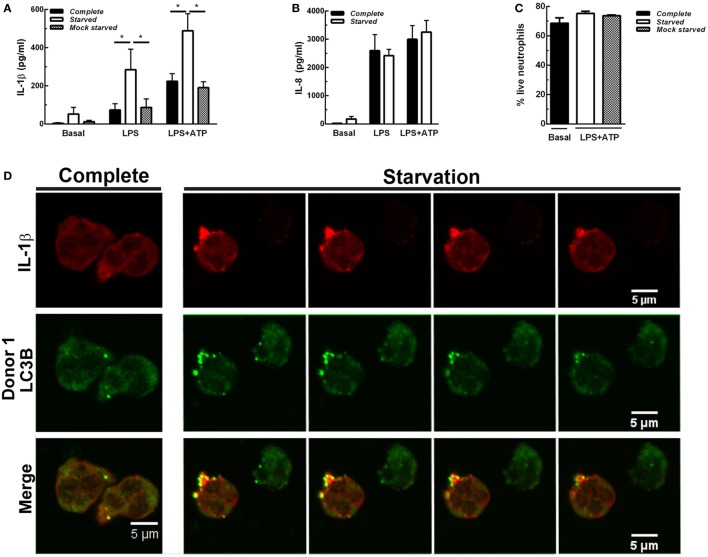

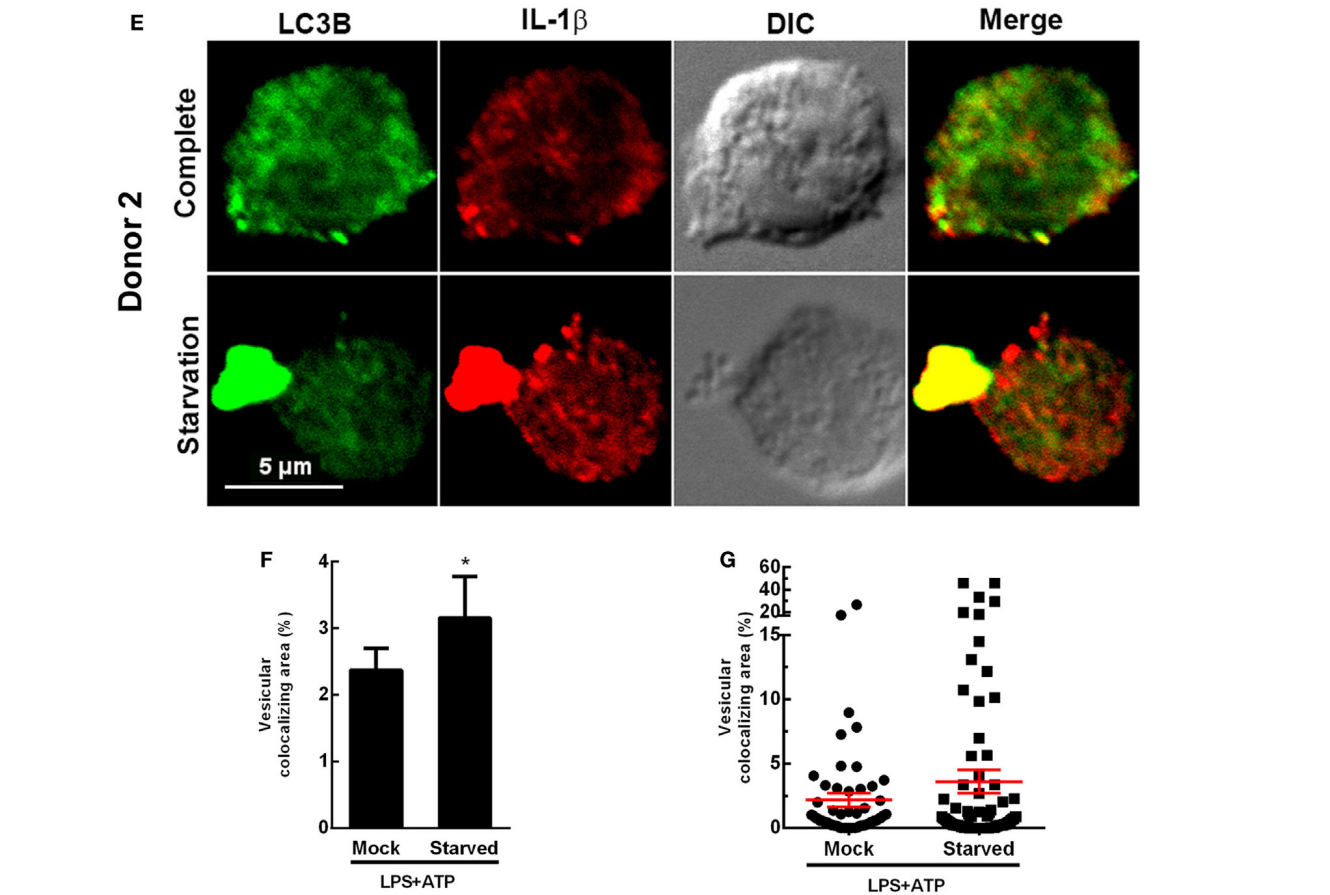
Effect of autophagy stimulation by starvation on neutrophil interleukin-1β (IL-1β) secretion. Neutrophils remained unstimulated (basal) or were stimulated with LPS and 2 h later were treated or not with ATP. At 3.5 h post-LPS stimulation, cells were left untreated (complete) or supernatants were removed and replaced by complete medium (mock starved) or Earle’s Balanced Salt Solution (starved) and cultured by an additional hour. Then, IL-1β **(A)** and IL-8 **(B)** concentrations in culture supernatants were determined. Graph depicts the mean ± SEM of four experiments performed in triplicate. * *p* < 0.01. **(C)** Viability of neutrophils treated as indicated. **(D,E)** Representative confocal laser scanning microscopy images of neutrophils stimulated with LPS + ATP for 3.5 h and then cultured in full nutrient medium (complete) or starvation medium (starvation) for 1 h. Cells were stained with specific antibodies anti-IL-1β (red) and LC3B (green). Images are representative of four experiments with different donors. [**(D)**; starvation panel] *Z* stack images that were collected at 0.30 µm intervals. **(F,G)** Quantifications of images of experiments depicted in **(D)**, performed by using a specific macro with Fiji software. **(F)** Data are expressed as the mean ± SEM of the percentage of the vesicular LC3B-IL-1β colocalizing area/total cell area of two independent experiments. **(G)** Scatter plot depicts the vesicular LC3B-IL-1β colocalizing area/total cell area of one of the experiments depicted in F. Red bars indicate the mean ± SEM values of 66 (mock) and 90 (starved) cells analyzed. *p < 0.05 (Mann–Whitney test analysis).

We then reasoned that if inhibition of autophagy reduced IL-1β secretion, stimulation of autophagy should promote IL-1β secretion. This fact was supported by results indicating that autophagy triggered by cell starvation markedly promoted IL-1β secretion induced both by LPS and LPS + ATP (Figure 4A). Of note, similar IL-1β levels were detected when cells were maintained in complete medium or centrifuged and suspended again in complete medium (mock starved), indicating that the procedure to which cells were subjected to undergo starvation did not affect IL-1β secretion. In additional assays we also determined that neutrophil that underwent starvation did not have a compromise in releasing IL-8 (Figure 4B), indicating that autophagy stimulation affects IL-1β secretion pathway but not the canonical protein secretion pathway. Of mention, this short starvation period did not modulate neutrophil viability (Figure 4C). In agreement with a role for autophagy in the translocation of cytosolic IL-1β to the extracellular medium, CLSM images of neutrophils stimulated by LPS + ATP evidenced a marked increment in the colocalization of IL-1β and LC3B upon starvation (Figure 4D; Video S1 in Supplementary Material). Noteworthy, we visualized in some cells wide autophagy vesicles upon starvation (Figure 4E). Moreover, we confirmed that starvation increased the autophagic vesicles containing IL-1β by image quantification of the vesicular area/cell with overlapping IL-1β- and LC3B-fluorescence signals (Figures 4F,G). Altogether, our results suggest that autophagy translocates IL-1β from the cytosol to the lumen of autophagosomes, and this mechanism is required for IL-1β secretion.

**Figure 5:**
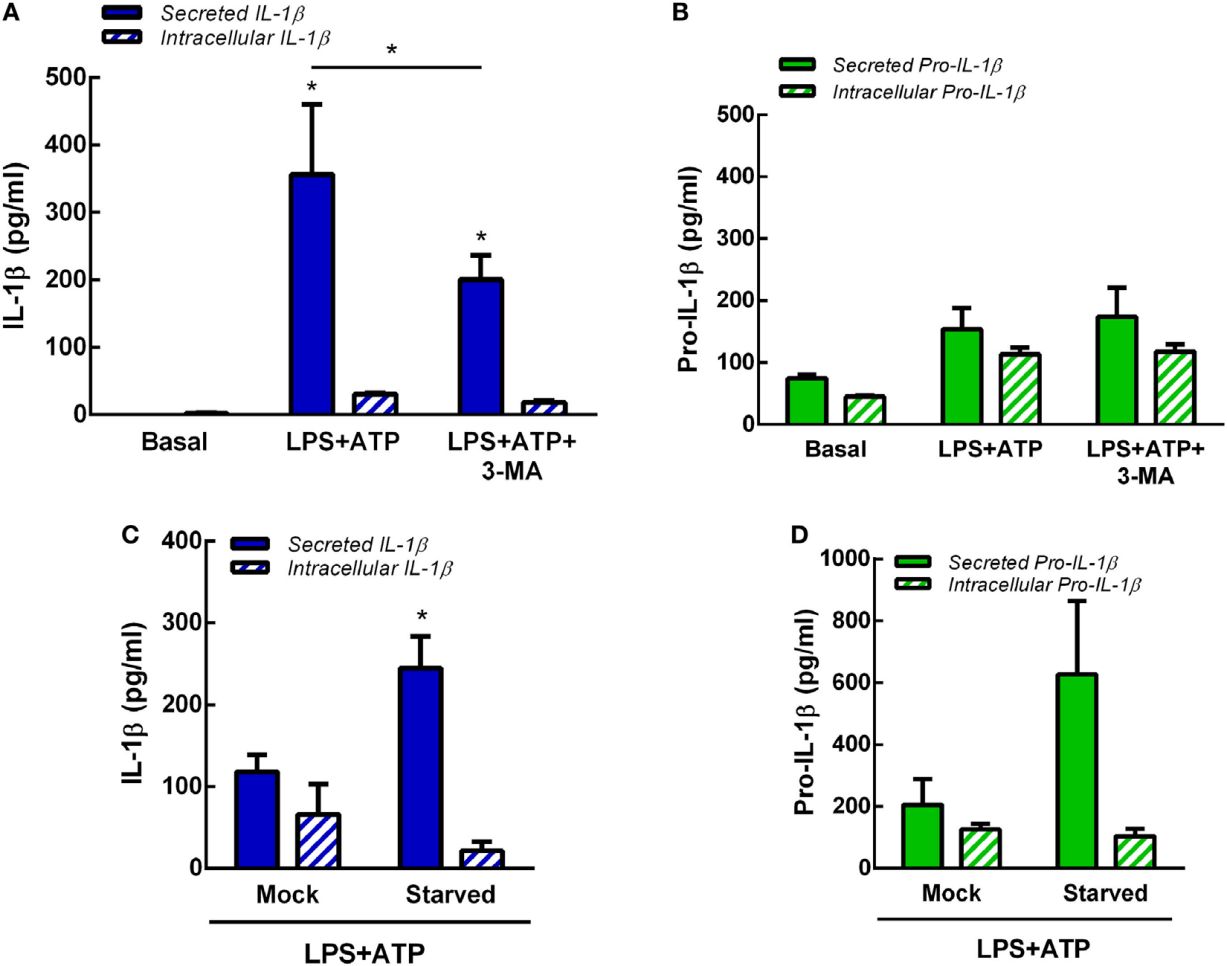
Role of autophagy in interleukin-1β (IL-1β) and pro-IL-1β secretion. Concentrations of IL-1β **(A)** and pro-IL-1β **(B)** in culture supernatants (secreted) and cell pellets (intracellular) of neutrophils stimulated or not with LPS + ATP for 5 h in the absence or presence of 3-methyladenine (3-MA) (5 mM) added 30 min before LPS stimulation. Concentrations of IL-1β **(C)** and pro-IL-1β **(D)** in culture supernatants (secreted) and cell pellets (intracellular) of mock-treated or starved neutrophils stimulated with LPS + ATP for 5 h. Data represent the mean ± SEM of four **(A,B)** or five **(C,D)** experiments performed in duplicate. Two-way ANOVA followed by Bonferroni’s multiple comparisons test.* *p* < 0.05 vs. basal or between LPS + ATP-treated or not with 3-MA.

In previous studies, we determined that at longer time points (18 h) not only IL-1β but also pro-IL-1β are released by human neutrophils upon LPS + ATP stimulation (7). Thus, to determine whether autophagy is involved also in pro-IL-1β secretion, we performed additional experiments by evaluating the effects of inhibition or promotion of neutrophil autophagy on both intracellular and secreted levels of pro-IL-1β and IL-1β by specific ELISAs. As shown in Figures 5A,B, both isoforms were released to culture supernatants at 5 h post-LPS + ATP stimulation. However, secreted pro-IL-1β levels were not statistically different to those observed under basal conditions. The inhibition of autophagy with 3-MA significantly reduced IL-1β secretion but did not modulate pro-IL-1β secretion. Moreover, 3-MA neither modulated intracellular IL-1β nor pro-IL-1β levels (Figures 5A,B). These results were in accordance to observations performed by flow cytometry (Figure S5 in Supplementary Material), which indicated that IL-1β intracellular levels exhibited by 3-MA-treated cells were similar to those observed in untreated cells at 5 h post-LPS + ATP stimulation. This unexpected lack of accumulation of IL-1β in 3-MA treated cells might be due to cytokine degradation at this time point. In additional assays we determined that stimulation of autophagy by starvation significantly promoted IL-1β secretion but not significantly modulate pro-IL-1β secretion (Figures 5C,D). Altogether, these data suggest that autophagy promotes IL-1β secretion but does not affect pro-IL-1β secretion.

**Figure 6:**
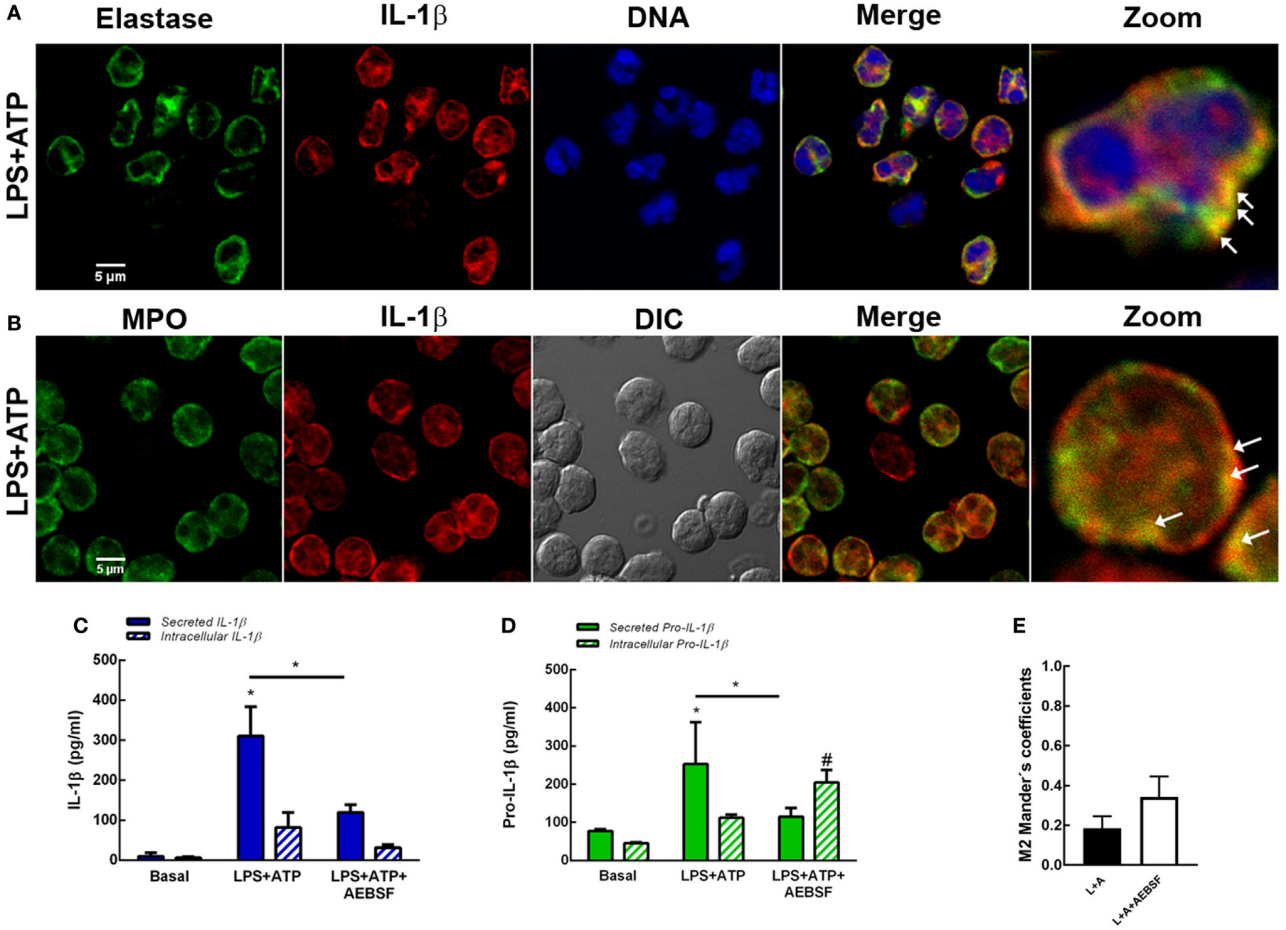
Role of serine proteases in interleukin-1β (IL-1β) secretion. Colocalization of IL-1β with elastase **(A)** or myeloperoxidase (MPO) **(B)**. Neutrophils were stimulated with LPS and 2 h later were treated with ATP. At 3.5 h post-LPS stimulation, cells were fixed, permeabilized, and stained with specific antibodies anti-IL-1β (red) and elastase [**(A)**; green] or MPO [**(B)**; green]. Images were acquired with a confocal microscope. Arrows indicate examples of compartments with IL-1β and elastase or MPO overlapping signals. Concentrations of IL-1β **(C)** and pro-IL-1β **(D)** in culture supernatants and cell pellets of neutrophils stimulated or not for 5 h with LPS + ATP in the absence or presence of AEBSF (1 mM) added 1 h post-LPS stimulation. Data represent the mean ± SEM of four experiments performed in duplicate. Two-way ANOVA followed by Bonferroni’s multiple comparisons test. * and ^#^ *p* < 0.05 vs. their respective basals or between LPS + ATP-treated or not with AEBSF. **(E)** Manders’ colocalization coefficients of IL-1β/LC3B for IL-1β (M2) from images of neutrophils stimulated 4 h with LPS + ATP and treated or not with AEBSF (1 mM) 1 h after LPS stimulation. Values were derived from five independent experiments with at least 60 cells analyzed per experiment. Results were evaluated by Mann–Whitney test analysis and differences were not statistically significant.

Neutrophils have four subsets of secretory granules (azurophilic, specific, and gelatinase granules; and secretory vesicles); and at least two other types of mobilizable organelles, the multivesicular bodies/late endosomes and recycling endosomes (21). Previous studies determined that autophagosomes can fuse to endosomes forming amphisomes (22). Other studies showed that granules occasionally fuse with each other in the cytosol prior to their subsequent fusion with the plasma membrane (23). Thus, taking into account that in previous studies we observed that Elafin, an elastase and PR3 inhibitor, partially reduced human neutrophil IL-1β release induced by LPS and LPS + ATP (7), we sought to determine whether serine proteases could modulate autophagy-mediated IL-1β secretion. We hypothesized that serine proteases-enclosing granules (azurophilic granules) could eventually fuse with vesicles containing IL-1β. To test this possibility, we analyzed if IL-1β could be found in vesicular compartments colocalizing with elastase. As shown in Figure 6A, we detected some IL-1β-containing vesicles colocalizing with elastase upon LPS + ATP stimulation. Similar colocalization was observed between IL-1β and myeloperoxidase (MPO), a marker of neutrophil azurophilic granules (21), the compartment where serine proteases are located in resting neutrophils (Figure 6B). According to an analysis performed with PROSPER software (24), the mature IL-1β sequence (aa116 to 269) contains multiple potential cleavage sites for neutrophil serine proteases (5 for elastase and 6 for cathepsin G; Figure S8 in Supplementary Material). Thus, we reasoned that serine proteases could modulate IL-1β levels to be secreted by either generating mature IL-1β or contributing to its degradation. To test these possibilities, we determined the effect of the serine protease inhibitor AEBSF on both the secreted- and intracellular IL-1β and pro-IL-1β levels and on IL-1β and LC3B colocalization. This compound significantly inhibited IL-1β and pro-IL-1β secretion at 5 h post-LPS + ATP stimulation (Figures 6C,D). AEBSF neither significantly modulated intracellular IL-1β levels (Figure 6C) nor intracellular pro-IL-1β levels upon LPS + ATP stimulation (Figure 6D), although a trend to an increase was observed in the last case. On the other hand, AEBSF did not significantly affect IL-1β/LC3B colocalization (Figure 6E).

## Discussion

Neutrophils are terminally differentiated short-lived cells that are refractory to transfection; thus, they are not amenable for gene silencing and fluorescence protein expression. Probably for these reasons information about human neutrophil vesicular trafficking and autophagy molecular machinery in these cells is scarce. On the other hand, the mechanisms involved in IL-1β exportation in neutrophils have not been examined so far. Thus, in this study we sought to investigate the role of autophagy in human primary neutrophil IL-1β secretion. We determined that autophagy is involved in human neutrophil IL-1β secretion induced by LPS and LPS + ATP. This conclusion is supported by our results showing that inhibition of both the induction of autophagy by 3-MA, Wortmannin and VPS34-IN1 as well as the blockade of the autophagic flux by Baf A1 and lysosomal degradation by E64d, strikingly reduced IL-1β release by human neutrophils. Furthermore, stimulation of autophagy by starvation significantly enhanced IL-1β secretion. Additional support for a role of autophagy in IL-1β secretion was provided by data indicating that ATG5 knockdown in neutrophil differentiated PLB985 cells markedly diminished IL-1β secretion. Our confocal microscopy studies could afford to confirm a role of autophagy in the translocation of cytosolic IL-1β to the extracellular medium, as indicated by the fact that IL-1β was found colocalizing with LC3B upon LPS stimulation at previous time points than those at which it was extracellularly found. These findings suggest that IL-1β is incorporated to an autophagosomal compartment, a fact that was markedly strengthen when LPS + ATP-stimulated neutrophils were subjected to starvation, as indicated by the increase in IL-1β/LC3B colocalization and the appearance of wide IL-1β+/LC3B+ vesicles. Our results also confirmed that both IL-1β and pro-IL-1β isoforms were released to culture supernatants upon LPS + ATP stimulation. However, only IL-1β secretion appears to be modulated by autophagy, as neither inhibition of autophagy with 3-MA nor promotion of autophagy by starvation modulated pro-IL-1β secretion. Altogether, these results suggest that IL-1β is incorporated to autophagic vesicles. These findings are in agreement with studies by Zhang et al. that showed in non-myeloid cells, that IL-1β enters the lumen of a vesicle, possibly a precursor of the phagophore, and that later turns into an autophagosome. The authors proposed that the IL-1β-containing autophagosomes may directly fuse with the plasma membrane or further fuse with an multivesicular bodies from which IL-1β is secreted (25).

Our studies also identified colocalization of vesicular IL-1β with elastase and MPO, suggesting that part of the vesicles containing IL-1β intersects azurophil granules content. Moreover, we confirmed that inhibition of serine proteases reduces both IL-1β secretion, as we have previously reported with Elafin (7), and pro-IL-1β secretion. However, inhibition of serine proteases did not significantly modulate either intracellular IL-1β levels or intracellular pro-IL-1β, although a trend to an increase was observed in the last case. Serine proteases inhibition did not significantly affect IL-1β/LC3B colocalization either. It might be possible that IL-1β-LC3B colocalization and IL-1β secretion are determined by a balance between the contribution of serine proteases and caspase-1 to pro-IL-1β processing, and the serine proteases impact on IL-1β degradation. Thus, the role of serine proteases in the control of IL-1β secretion is more complex than would have been expected. Further studies are required to conclude about the role of serine proteases in IL-1β processing and autophagy-mediated secretion.

Several studies have dealt with the mechanisms involved in IL-1β secretion in macrophages, monocytes, and dendritic cells. However, disparate observations have been reported probably arising from different experimental systems. Our studies partially agree with those reported by Dupont et al., which indicated that induced autophagy ferries IL-1β from the cytosol into the autophagosome, and by doing so, to the extracellular medium in mouse bone-marrow-derived macrophages (BMM) stimulated with LPS plus different inflammasome agonists (15). In fact, as we observed in human neutrophils, the authors showed that both starvation and pharmacological induction of autophagy by mTOR inhibition with pp242 increased secretion of IL-1β. Moreover, they also determined that blocking of autophagy maturation with Baf A1 inhibited IL-1β secretion. In contrast to Dupont et al.’s study, which demonstrated that basal autophagy negatively affected IL-1β secretion in BMM; basal autophagy in neutrophils (unstimulated cells) did not modulate IL-1β secretion. Our findings are also in accordance with those indicating that inhibition of autophagy with 3-MA or by siRNA-mediated Beclin-1 inhibits IL-1β secretion in human macrophages that have been activated through the Dectin-1 pathway (26).

By contrast, other studies indicated that ATG16L1 deficiency, which severely impairs autophagosome formation, increases IL-1β secretion by mouse macrophages stimulated with LPS, indicating that inhibition of basal autophagy induces IL-1β overproduction (27). In the same line, additional work determined that inhibition of autophagy with 3-MA and Wortmannin-induced IL-1β secretion by mouse BMM and dendritic cells stimulated with LPS (28). These studies also found that stimulation of autophagy with rapamycin inhibited IL-1β secretion induced either by LPS or LPS plus inflammasome activation stimuli like ATP, chitosan, and alum. The authors concluded that autophagy acts to limit the availability of pro-IL-1β within stimulated mouse macrophages and dendritic cells. The reasons for differences with our findings are not understood; however, it should be considered that we have previously demonstrated the existence of differences in IL-1β processing and secretion between human neutrophils and mouse macrophages. In fact, this study found, in contrast to what we previously determined in human neutrophils (7), that IL-1β secretion induced by LPS + ATP by mouse macrophages did not require NOX2 activity.

Our findings also contrast with those reported by Nakahira et al., who determined that depletion of the autophagic proteins LC3B and Beclin-1 enhanced the secretion of IL-1β and IL-18 by mouse BMM. These authors determined that disruption of LC3B generates a defect in mitochondrial homeostasis, which results in a greater basal amount of mitochondrial ROS production and renders mitochondria more susceptible to damage by treatment with LPS and ATP. This, in turn, results in mitochondrial DNA release and enhanced caspase-1 activation and higher levels of IL-1β secretion (29). However, in human blood neutrophils, mitochondria not only are scarce but also are different from those of other myeloid cells, because they preserve mainly death-mediating abilities. Neutrophils mainly use glycolysis rather than mitochondrial oxidative phosphorylation for their energy supply, and mitochondria hardly participate in ATP synthesis. Moreover, neutrophils have 10–15 times less copies of the mitochondrial genome in comparison to peripheral blood mononuclear cells (30). Thus, differences in the impact of autophagy in IL-1β secretion between Nakahira et al.’s and our findings could rely on the peculiarities of the physiology of both cell systems.

Other studies also indicated that autophagy limits IL-1β production by targeting ubiquitinated inflammasomes for destruction in differentiated human THP-1 cells (31). The authors also found that starvation inhibited IL-1β secretion and autophagy inhibition promoted IL-1β secretion in human monocytes primed with LPS and then transfected with poly(dA:dT) or treated with nigericin to stimulate inflammasome activation. Although the consequences of autophagy modulation on IL-1β secretion contrast with those we report here for human neutrophils, it is possible to speculate that exist differences between inflammasome stability in neutrophils and monocytes. In fact, we previously reported that neutrophil NLRP3 expression is not modulated by LPS priming in contrast to the observation we made in human monocytes (7) and to what was reported for mouse macrophages (32).

In contrast to these findings, recent studies in differentiated THP-1 cells have identified distinctive molecular components involved in secretory autophagy that distinguishes it from degradative autophagy. These studies indicated that in response to LPS plus lysosomal damage or LPS plus starvation, intracellular IL-1β is recognized by the tripartite motif protein 16 (TRIM16) which in turn interacts with the R-SNARE Sec22b to deliver IL-1β to autophagic membranes. The authors also provided evidence for a role of certain SNAREs (syntaxin 3, syntaxin 4, SNAP23, and SNAP29) in membrane fusion of the autophagic vesicles containing IL-1β with the plasma membrane (33, 34). Whether these molecular mechanisms are involved IL-1β secretion mediated by secretory autophagy in human neutrophils remains to be determined. Nevertheless, it is interesting to mention that studies performed in differentiated HL-60 cells have shown that siRNA-mediated knockdown expression of syntaxin 3 reduces IL-1β secretion induced by LPS (35).

Beyond the differences between species and properties of distinct myeloid cells that could be responsible for disparate roles of autophagy in the control of IL-1β secretion, it is noteworthy that even in the same cell type (differentiated THP-1, for example) secretory and degradative roles for autophagy have been reported. Thus, as has been previously suggested (33), the utilization of autophagy for regulated secretion of IL-1β might provide a cell with the opportunity to throttle inflammatory outputs by promoting its secretion (15) or reducing its levels either directly (28), or by means of trimming down the inflammasome components (31) or eliminating endogenous inflammasome agonists (29, 36).

In conclusion, our study adds to the understanding of the mechanisms that control IL-1β secretion in human neutrophils. Considering the diverse infectious and inflammatory conditions where neutrophils infiltrate the tissues in large numbers, these findings could contribute to identify potential targets to control IL-1β-mediated inflammation in those diseases where neutrophil-derived-IL-1β plays a crucial role in their pathogenesis.

## Author Contributions

LI and IK designed the experiments, carried out most of them and analyzed data; FS, PPG and MG conducted some experiments; FF programmed the image quantification macros, and performed microscopy acquisitions together with LI, IAK and AST; JG, MO, JRG and CCJ provided scientific expertise, analyzed and interpreted data and wrote the manuscript; AST conceived the research, analyzed and interpreted data and wrote the manuscript. All authors reviewed the manuscript.

## Conflict of Interest Statement

The authors declare that the research was conducted in the absence of any commercial or financial relationships that could be construed as a potential conflict of interest.
