# Supplementary material for: Autophagy Mediates Interleukin-1β Secretion in Human Neutrophils
Source: Front Immunol. 2018 Feb 19;9:269. doi: 10.3389/fimmu.2018.00269 (PMC5825906; doi:10.3389/fimmu.2018.00269)
Supplement: Supplementary file 1 [file data_sheet_1.PDF]

Supplementary Material

Autophagy mediates Interleukin-1β secretion in human neutrophils

Leonardo Iula[1#], Irene A. Keitelman[1#], Florencia Sabbione[1], Federico Fuentes[1], Mauricio Guzman[1], Jeremías Galletti[1], Pehuén Pereyra Gerber[2], Matías Ostrowski[2], Jorge R. Geffner [2,3], Carolina C. Jancic[1,3], and Analía S. Trevani[1,3*]

*** Correspondence:** Corresponding Author: analiatrevani@yahoo.com.ar/analiatrevani@gmail.com

1 Supplementary Data

2 Supplementary Figures and Tables

2.1 Supplementary Figures

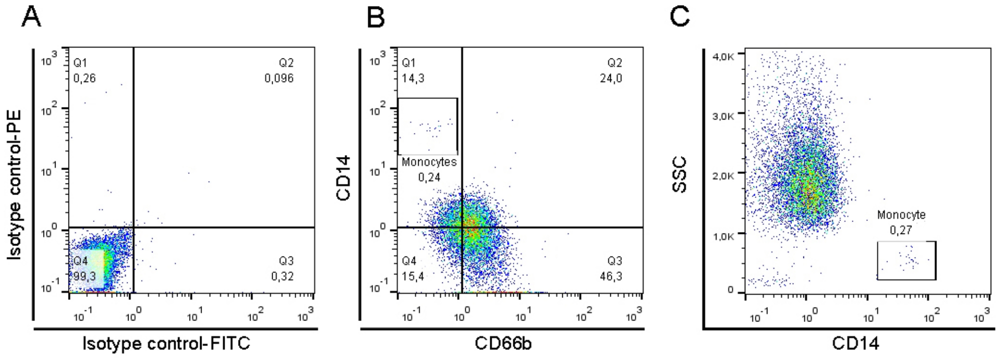

Figure S1. Purity of neutrophil preparations. Representative dot plots of the isolated neutrophil populations. Dot plots of isotype controls (A), CD14 vs CD66b expression (B) and SSC-H vs CD14 expression (C). Gated cells in B (0.24%) and C (0.27%) represent the contaminating monocytes ($CD14^{high}$ population). As positive staining of neutrophils with CD66b and CD14 yielded similar results, only CD14 expression was used throughout the manuscript to determine monocyte contamination of neutrophil preparations.

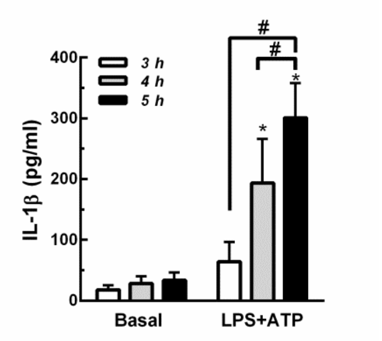

Figure S2. Kinetics of neutrophil IL-1β secretion induced by LPS+ATP. Neutrophils were stimulated with LPS (250 ng/ml) and 2 h later were treated with ATP (2.5 mM). At 3-, 4- and 5 h post-LPS stimulation supernatants were collected and IL-1β concentrations were determined by ELISA. Two-way ANOVA followed by Bonferroni's multiple comparisons test *p<0.05 vs basal; #p<0.05.

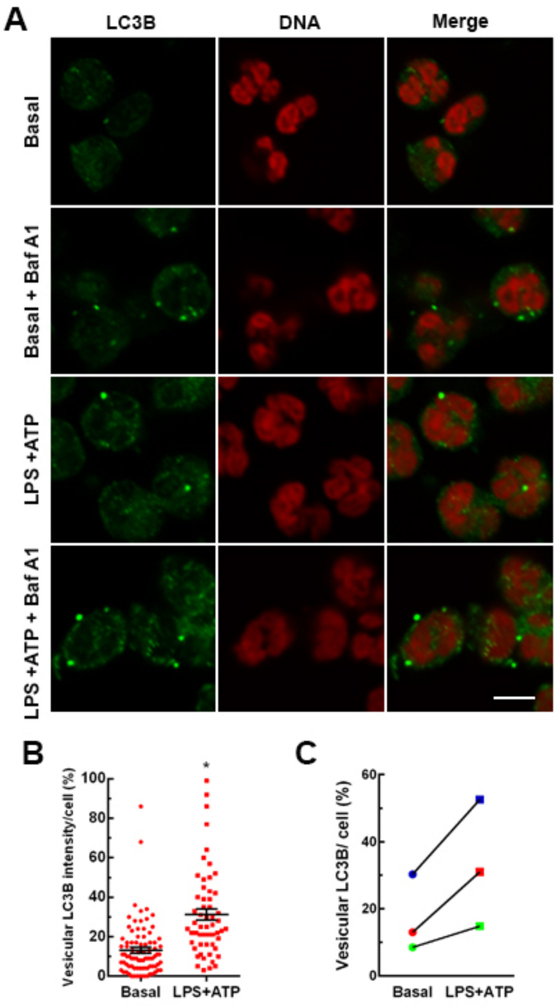

Figure S3. Stimulation with LPS+ATP promotes neutrophil autophagy. Neutrophils were stimulated with LPS (250 ng/ml), 2 h later were treated or not with ATP (2.5 mM) and 15 min later with Baf A1(100 nM). At 3 h post-LPS stimulation, cells were fixed, permeabilized and stained with a specific antibody anti-LC3B (green) and DNA was stained with TO-PRO-3 (red). Images were acquired with a confocal microscope (A) and quantifications (B and C) were performed by using a specific macro with Fiji software. (A) Representative images of three different experiments. Bar: 5 µm. (B) Scatter plot depicts the percentage of vesicular LC3B intensity/cell from a representative experiment of two. Black bars indicate the mean ± SEM values of 84 (basal) and 56 (LPS+ATP) cells analyzed. *P<0.05 (Mann Whitney test analysis). (C) Data represent the mean value of vesicular LC3B intensity/cell from experiments like that depicted in B (red symbols), performed with three different donors in which at least 35 cells were analyzed for each experiment.

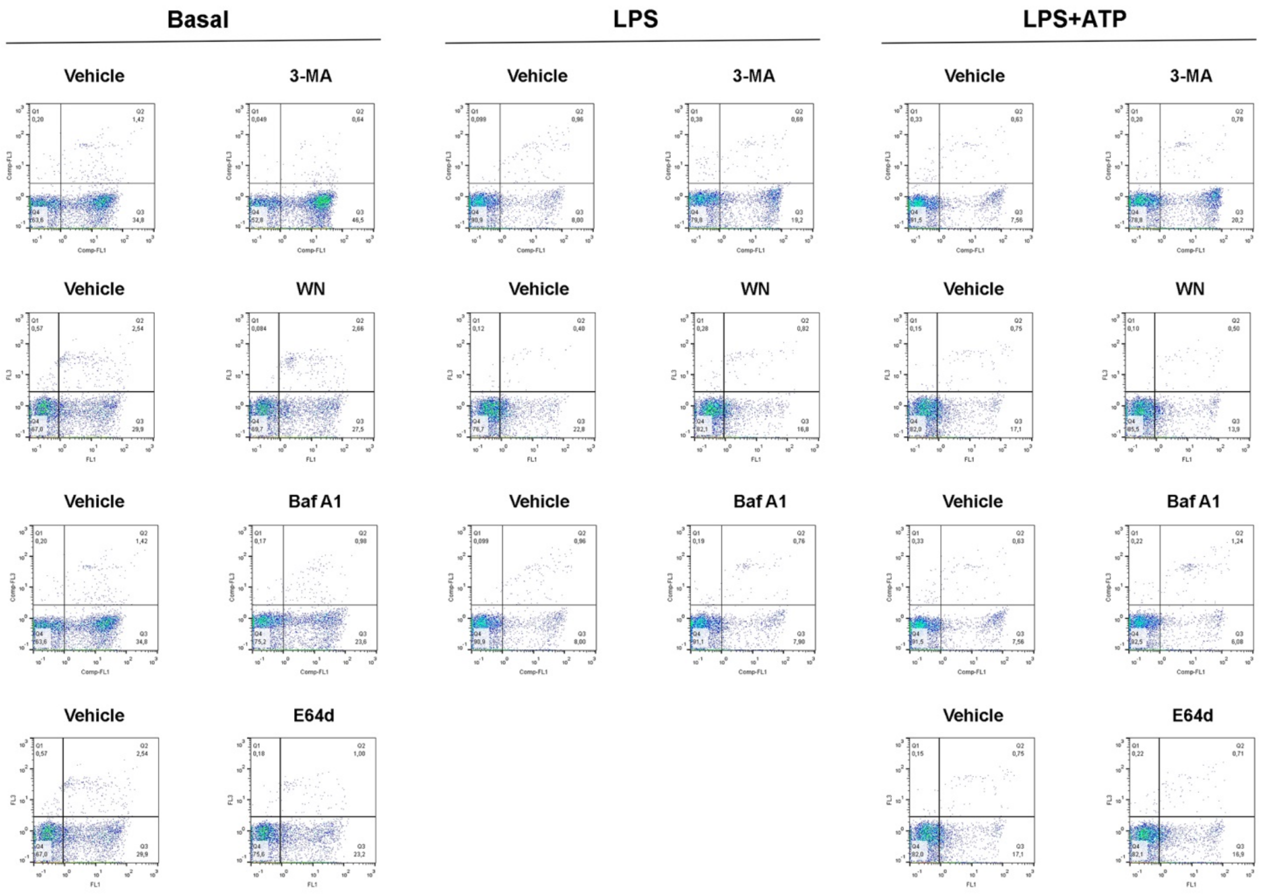

Figure S4. Representative dot plots of viability assays of neutrophils left unstimulated (basal) or stimulated with LPS or LPS+ATP and subjected or not (vehicle) to treatment with the indicated autophagy inhibitors, evaluated by annexin V/propidium iodide staining and flow cytometry.

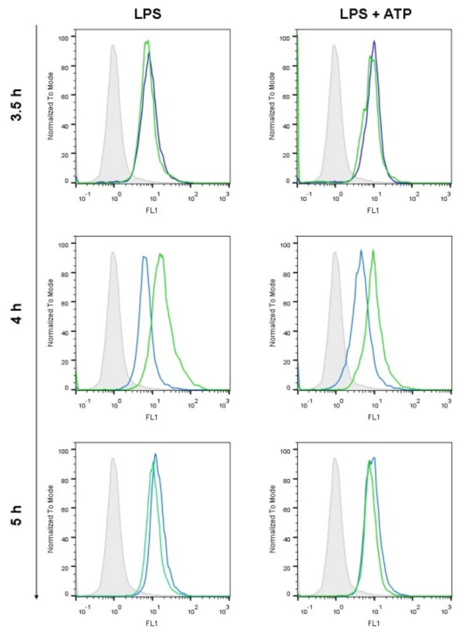

Figure S5. 3-MA did not inhibit IL-1β synthesis. Neutrophils were pretreated or not with 3-MA (5 mM) and then stimulated with LPS or LPS+ATP. At 3-, 4-, or 5 h post-LPS stimulation, cells were fixed, permeabilized and after staining with a specific antibody, IL-1β expression was evaluated by flow cytometry. Histograms are representative of one donor of 4 evaluated. Isotype controls, tinted grey histograms; neutrophils stimulated in the absence (blue histograms) or the presence (green histograms) of 3-MA.

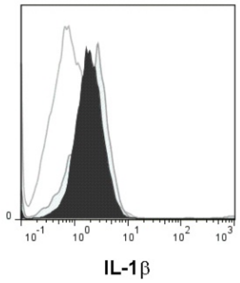

Figure S6. Histograms represent IL-1β expression in scramble- (Scr; light grey tinted) or Atg5-siRNA transfected (dark grey tinted) neutrophil-differentiated PLB985 cells stimulated with LPS+ATP at 3 h post LPS-stimulation. Grey line: isotype control.

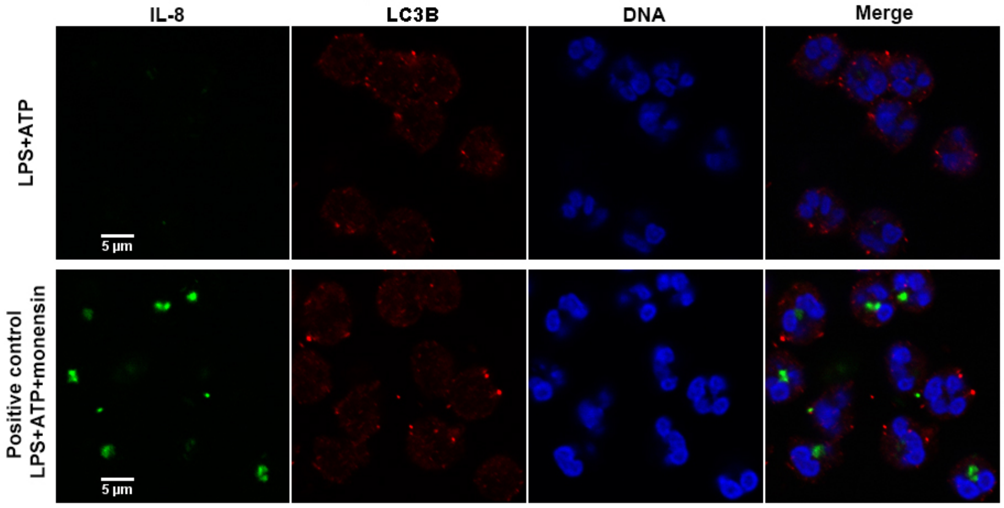

Figure S7. Interleukin-8 (IL-8), a prototype of conventionally secreted cytokine, did not colocalize with LC3B. Neutrophils were stimulated with LPS (250 ng/ml), in the absence (top) or presence (bottom) of monensin and 2 h later were treated or not with ATP (2.5 mM). At 4 h post-LPS stimulation, cells were fixed, permeabilized and stained with a specific antibody anti-IL-8 (green), anti-LC3B (red) and DNA was stained with TO-PRO-3 (blue). Images were acquired with a confocal microscope.

A

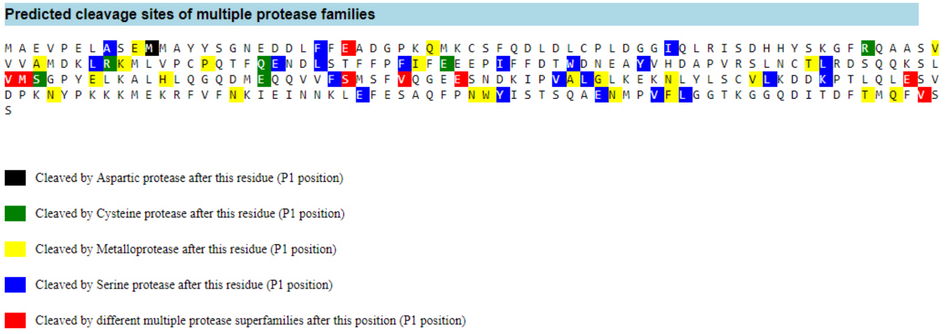

B

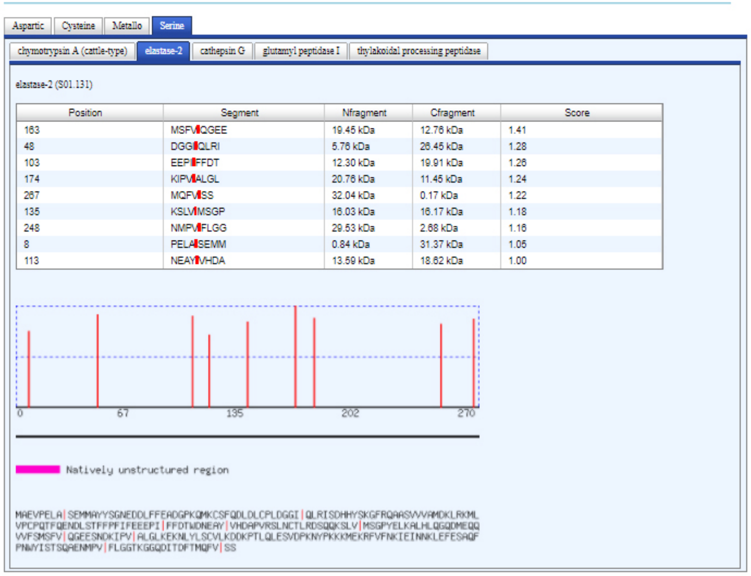

Position	Segment	Nfragment	Cfragment	Score
163	MSFVQGEE	19.45 kDa	12.76 kDa	1.41
48	DGGIQLRI	5.76 kDa	26.45 kDa	1.28
103	EEPIFFDT	12.30 kDa	19.91 kDa	1.26
174	KIPVALGL	20.76 kDa	11.45 kDa	1.24
267	MQFVSS	32.04 kDa	0.17 kDa	1.22
135	KSLVMSGP	16.03 kDa	16.17 kDa	1.18
248	NMPVFLGG	29.53 kDa	2.68 kDa	1.16
8	PELASEMM	0.84 kDa	31.37 kDa	1.05
113	NEAYVHDA	13.59 kDa	18.62 kDa	1.00

C

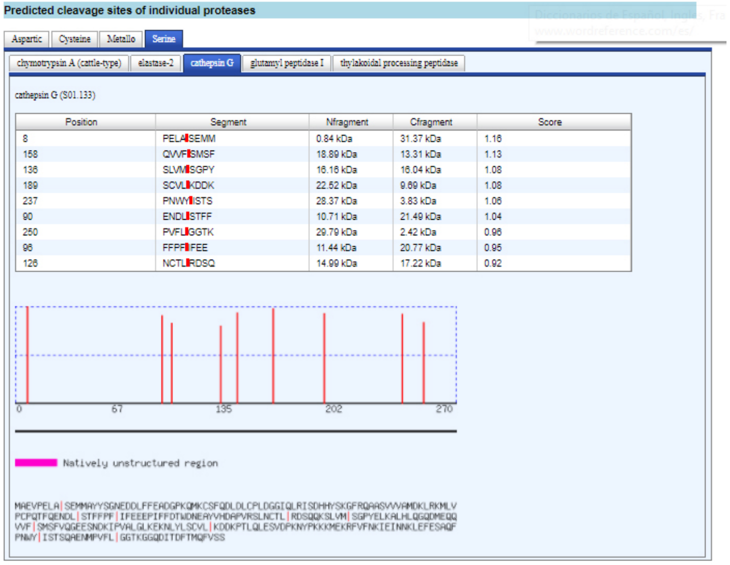

Position	Segment	Nfragment	Cfragment	Score
8	PELASEMM	0.84 kDa	31.37 kDa	1.16
158	QVVFSMSF	18.89 kDa	13.31 kDa	1.13
136	SLVMSGPY	16.16 kDa	16.04 kDa	1.08
189	SCVLKDDK	22.52 kDa	9.69 kDa	1.08
237	PNWYISTS	28.37 kDa	3.83 kDa	1.06
90	ENDLSTFF	10.71 kDa	21.49 kDa	1.04
250	PVFLGGTK	29.79 kDa	2.42 kDa	0.96
96	FFPFIFEE	11.44 kDa	20.77 kDa	0.95
126	NCTLRDSQ	14.99 kDa	17.22 kDa	0.92

Figure S8. Predicted cleavage sites in pro-IL-1β sequence for multiples proteases (A) and those for elastase (B) and cathepsin G (C) calculated with PROSPER software (https://prosper.erc.monash.edu.au).

3 Legend to Supplementary Video

Supplementary Video. 3D reconstruction of a representative human neutrophil of those stimulated with LPS (250 ng/ml), 2 h later treated with ATP (2.5 mM) and at 3.15 h post-LPS stimulation, subjected to starvation for 90 minutes. Sequential projections in the Y-axis from a stack of 1.2 μm interval images, acquired using a laser scanning confocal microscope. Cells were immunolabeled with antibodies against IL-1β (red) and LC3B (green).
